# NUCB2 promotes hepatocellular carcinoma cell growth and metastasis by activating the E2F4/PTGR1 axis

**DOI:** 10.7150/ijbs.97861

**Published:** 2024-09-03

**Authors:** Yuan Wang, Bin Sun, Wei Wei, Tao Han, Jing Ma, Xiaodong Li, Chuanchun Han, Zhikun Lin

**Affiliations:** 1 The Second Affiliated Hospital, Dalian Medical University, Dalian, Liaoning 116044, P.R. China.; 2 Institute of Cancer Stem Cell, Dalian Medical University, Dalian, Liaoning 116044, P.R. China.; 3 The First Affiliated Hospital of Dalian Medical University, Dalian 116044, P.R. China.; 4 College of Medical Laboratory, Dalian Medical University, Dalian 116044, P.R. China.; 5 College of Health-Preservation and Wellness, Dalian Medical University, Dalian, Liaoning 116044, P.R. China.

## Abstract

**Background:** The important role of nucleobindin 2 (NUCB2) in various cancers has been recently recognized. However, its biological functions and regulatory mechanisms in hepatocellular carcinoma (HCC) remain unclear.

**Methods:** The expression level of NUCB2 in HCC was assessed using public databases, immunohistochemistry, and Western blotting. The effects of NUCB2 on cell proliferation and metastasis were investigated using colony formation, EdU, Transwell assays, and an *in vivo* mouse xenograft model. Regulation of E2F4 by NUCB2 was identified by protein half-life and *in vivo* ubiquitylation assays. The relationship between E2F4 and prostaglandin reductase 1 (PTGR1) was investigated by qRT-PCR, RT-PCR, and chromatin immunoprecipitation assays.

**Results:** This study found that NUCB2 expression was significantly higher in HCC tissues than in normal liver tissues, and patients with high expression displayed shorter survival rates. Inhibition of NUCB2 reduced the proliferation and metastatic potential of HCC cells *in vitro* and *in vivo*. NUCB2 depletion reduced PTGR1 expression, which reduced cell proliferation and migration. Our findings suggested that NUCB2 suppressed E2F4 degradation by interacting with E2F4. Additionally, increased E2F4 levels facilitated PTGR1 transcription by directly binding to the PTGR1 promoter.

**Conclusion:** This study demonstrated the oncogenic properties of NUCB2 in HCC and suggested that NUCB2 facilitates hepatocellular carcinoma progression by activating the E2F4/PTGR1 axis.

## Background

Hepatocellular carcinoma (HCC) is a main type of liver cancer, being 85%-90% of all liver cancers^1^, ^2^. It is estimated that 70%-80% of HCC patients are diagnosed at a late period, and the five-year survival rate is only 12%, much lower than the survival rate for other types of cancer^3^. Traditional treatments, such as surgical resection, transplantation, radiofrequency ablation, and chemotherapy, are unsatisfactory in advanced HCC^4^, ^5^. Therefore, uncovering the molecular mechanisms underlying HCC and establishing effective therapeutic strategies are crucial.

Human nucleobindin 2 (NUCB2), is primarily found in 1994 in acute lymphoblastic leukemia cells 6. NUCB2 is involved in multiple physiological functions, including food intake, energy homeostasis, and cardiovascular and immune regulation^7^-^9^. Several studies have reported that NUCB2 plays an essential oncogenic role in many cancers including breast^10^, ^11^, colon^12^, skin^13^, ^14^, lung^15^, ^16^, renal^17^, glioblastoma^18^ and prostate cancers^19^. NUCB2 expression was found to be increased in ER-and PR-positive breast cancers, promoting cancer cell metastasis^10^, ^11^. In colon cancer, high NUCB2 expression enhanced cell migration and growth^12^. Several studies demonstrated that NUCB2 suppressed the progression of ovarian cancer and adrenocortical carcinoma^20^, ^21^. Elevated NUCB2/nesfatin-1 expression in the ovarian epithelial cell carcinoma cell line HO-8910 resulted in G1/S phase arrest of the cell cycle, advancing cell apoptosis^20^. In adrenocortical carcinoma, elevated NUCB2/nesfatin-1 levels induced the expression of the pro-apoptotic protein Bax^21^. The role of NUCB2/nesfatin-1 in tumorigenesis appears to be dual. However, the biological function of NUCB2 and its regulatory mechanisms in HCC remain unclear.

The present study showed that NUCB2 expression was notably higher in HCC tissues than in normal liver tissues. Patients with high levels of NUCB2 expression exhibited a poor prognosis. NUCB2 depletion significantly reduced HCC cell proliferation and metastasis, both *in vitro* and *in vivo*. Subsequently, NUCB2 depletion downregulated prostaglandin reductase 1 (PTGR1) expression and suppressed cell proliferation and migration. Furthermore, NUCB2 inhibited E2F4 degradation by interacting with it. Increased E2F4 facilitated the transcription of PTGR1 by directly binding to the promoter of PTGR1. Taken together, this study highlights the oncogenic properties of NUCB2 in HCC and suggests that NUCB2 enhances HCC progression by activating the E2F4/PTGR1 axis.

## Materials and Methods

### Cell culture and reagents

Hepatocellular carcinoma cell line Huh-7 was cultured using the DMEM medium. The RPMI1640 and MEM mediums were used to culture SNU449 cells. The antibodies were used: GAPDH (SC-25778, 1: 1000), Ubiquitin antibody (#3933S, 1: 2000), NUCB2 antibody (AB229683, 1: 3000), E2F4 (40291S, 1: 1000), PTGR1 (Proteintech, 13374-1-AP, 1: 6000), Ki67 antibody (Abcam, ab15580, 1: 500), Cleaved Caspase 3 antibody (Cell Signaling Technology, #9661, 1: 400), CD31antibody (1265-1-AP, 1: 300).

### Cell transfection and gene knockdown

In our study, we inserted shRNA sequences of NUCB2, E2F4 and PTGR1 into the pLKO.1-puro vector. The shRNA sequences were listed: human NUCB2#1: 5-GCTGGAATATCATCAGGTCAT-3 and NUCB2#2: 5-CCAGGAAGCAAAGATCAACTA-3; E2F4#1: 5-CCCTCTCTTCATTTCGGCTTT-3 and E2F4#2: 5-GACCTCTTTGATGTGCCTGTT-3; PTGR1#1: 5-CTTGGATTTGATGTCGTCTTT-3 and PTGR1#2: 5-GACTTGCTGAAATGGGTCTTA-3 and mouse NUCB2 5-CCACCAGAATCCTAACACATT-3.

### RT-qPCR assay and RT-PCR assay

Invitrogen's Trizol (Invitrogen) was used to extract the total RNA, followed by PrimeScriptTM RT reagent kit (Takara, RR047A) for cDNA synthesis. The followed primers were used. β-actin Forward: 5-CACCTTCTACAATGAGCTGCGTGTG-3 and Reverse: 5-ATAGCACAGCCTGGATAGCAACGTAC-3, and PTGR1 Forward: 5-ATATAACAGAACCGGCCCAC-3 and Reverse: 5-CCCTCTAAGACCCATTTCAGC-3, PLAC8 Forward: 5-TCTCTACAGGACCCGATATGG-3 and Reverse: 5-TTAGAAAGTACGCATGGCTCTC-3, CELF6 Forward: 5-TTTGGAGCCGTTGTCTCTG-3 and Reverse: 5-GAGCCTCTTCATGCCAATTTG-3, GLS2 Forward: 5-TCCACAACTATGACAACCTGAG-3 and Reverse: 5-GCTGAGACATCGCCACTATAG-3.

### Colony formation and cell migration assays

The colony formation assay was performed by inoculating Hepatocellular carcinoma cells into a 6-well plate. Huh-7 and SNU449 cells with or without NUCB2 or PTGR1 depletion were cultured at 37 °C within an incubator containing 5% CO_2_ for one week. After 15 minutes of methanol fixation, cells were washed with PBS and cllular samples were stained for 10 minutes with a 0.1% solution of crystal violet stain. Pictures of the colonies were obtained.

To measure Hepatocellular carcinoma cell migration, 40,000 cells were added to 24-well Transwell plates containing 8mm polyethylene terephthalate filters.

### Chromatin immunoprecipitation (ChIP) assay and dual-luciferase assay

An EZ-ChIP kit (17-371, Millipore) was used to conduct the ChIP assay using the anti-E2F4 antibody in the SNU449 cells.

The PTGR1 promoters were inserted into a pGL3-basic vector. The Dual-Luciferases Kit was used to measure the PTGR1 promoter's luciferase activity.

### RNA sequencing analysis and label-free quantitative proteomics

SNU449 cells with and without NUCB2 knockdown were sent to RNA sequencing analysis in BioMaker (Beijing, China). For quantitative proteomics, 10^6^ SNU449 cells with and without NUCB2 knockdowns were sent to Jingjie PTM Biolab to identify the altered proteins.

### *In vivo* tumor formaton and metastasis assays

1 × 10^7^ Huh-7 cells/mouse were injected subcutaneously into NOD SCID female mice. Tumor size was measured at regular intervals of 2 days, beginning at 6 days after injection. The animals were executed at 20 days post-injection. The tumor weight and volume were counted.

3 × 10^6^ Hepa1-6 cells/mouse were injected into C57BL/6J mice via lateral tail vein. After 51 days, the mice were killed, and lungs of the mice were fixed using a 4% formalin. Metastatic nodes were assessed by histological analysis.

### Bioinformatics analysis

TNMplot (https://tnmplot.com) database was used to analyze the expression of NUCB2 in371 primary Hepatocellular carcinoma tissue and 225 normal tissues. The survival rates were obtained from Kaplan-Meier plotter and GEPIA database.

### Statistical analyses

Statistically significant differences were investigated using GraphPad Prism 8. Comparisons of two groups were analyzed using Student's t-test. The correlation analysis used Spearman's correlation. P < 0.05 was defined as a significant difference.

## Results

### NUCB2 was significantly increased in HCC

**Figure 1:**
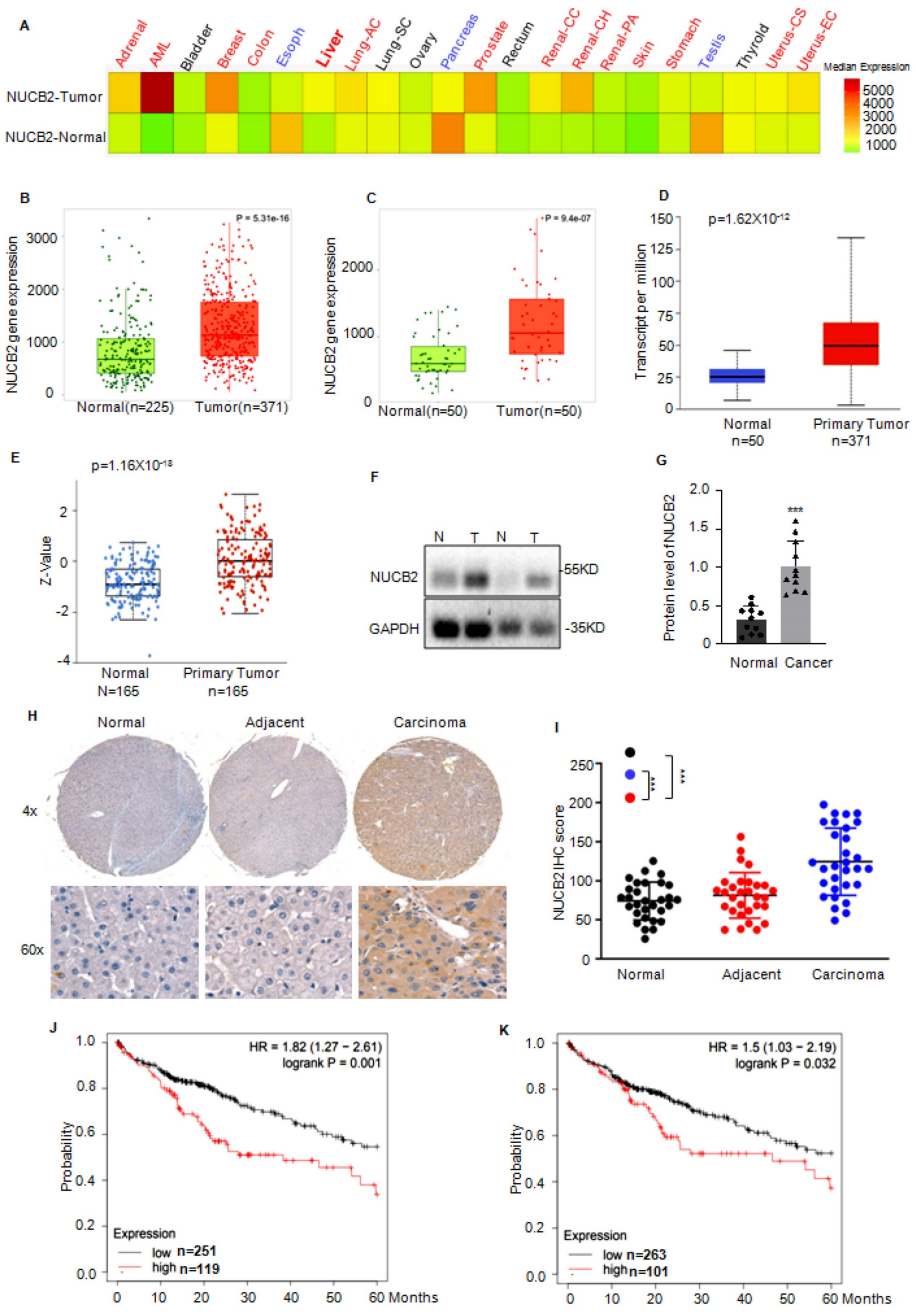
(A) The pan-cancer analysis indicated NUCB2 expression in normal and tumor tissues. (B) The TNMplot database was used to analyze NUCB2 expression levels in 371 hepatocellular carcinoma patients and 225 cases of normal tissue. (C) The TNMplot database was used to analyze NUCB2 expression levels in paired tumors and adjacent normal tissues (n = 50). (D) The UNLCAN database was used to analyze NUCB2 expression in 371 primary tumors of hepatocellular carcinoma and 50 normal tissues. (E) The UNLCAN database was used to analyze the protein levels of NUCB2 in 165 primary tumors of hepatocellular carcinoma and 165 normal tissues. (F) Western blotting was used to detect the protein levels of NUCB2 in hepatocellular carcinoma tissues and adjacent tissues (n = 11). The data were analyzed by Student's t-test, *p < 0.05, **p < 0.01, and ***p < 0.001. (H-I) Immunohistochemical staining of normal liver, adjacent, and hepatocellular carcinoma tissues (n = 30), followed by evaluation of NUCB2 expression. (J) Kaplan-Meier plot of the overall survival rate of 370 patients with hepatocellular carcinoma. The data were obtained from the Kaplan-Meier Plotter. (K) Kaplan-Meier plot of the overall survival rate of 364 patients with hepatocellular carcinoma. The data were obtained from Kaplan-Meier Plotter.

Initially, the expression levels of NUCB2 in tumors from different tissues were investigated to elucidate its function in liver cancer, and NUCB2 was increased in liver cancer (Figure 1A). Compared to the adjacent normal tissue, the RNA levels of NUCB2 were also significantly upregulated in the paired tumors (Figures 1B-C). Similar results were obtained from the UALCAN database that NUCB2 expression levels were notably elevated in HCC tissues (Figures 1D-E). To confirm this finding, we assessed NUCB2 expression by Western blotting and immunohistochemistry (IHC) using fresh HCC and adjacent tissues or serial sections of tissue microarrays. Consistently, NUCB2 protein levels were dramatically increased in the HCC tissues (Figures 1F and I). Patients with high NUCB2 expression displayed shorter survival rates than those with low NUCB2 expression (Figures 1J-K). Collectively, our results indicated that NUCB2 was markedly increased in HCC tissues.

### NUCB2 inhibition reduced HCC cell growth and metastasis *in vitro* and *in vivo*

**Figure 2:**
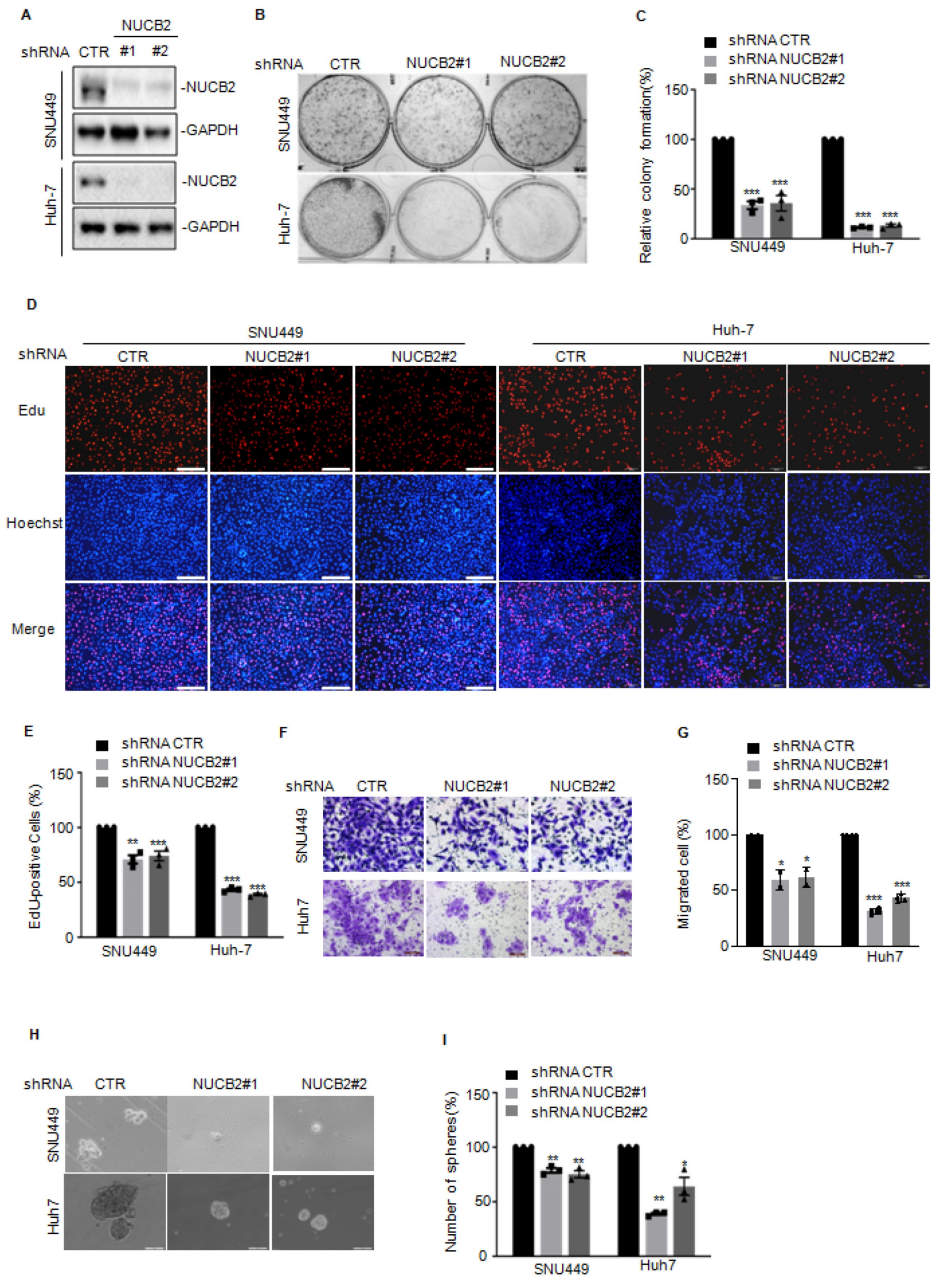
(A) Western blotting was used to analyze the NUCB2 expression in SNU449 and Huh-7 cells with or without NUCB2 knockdown. (B-C) The colony formation assay was used to investigate the effect of NUCB2 knockdown on hepatocellular carcinoma cell growth. (D-E) EdU assay was used to determine the impact of NUCB2 depletion on cell proliferation. (F-G) Transwell assay was used to measure the indicated cell migration. (H-I) Sphere formation assay measured the cancer stem cell property of hepatocellular carcinoma cells with or without NUCB2 knockdown. The C, E, G, and I data were analyzed by Student's t-test, *p < 0.05, **p < 0.01, and ***p < 0.001.

We then investigated the biological function of NUCB2 in liver cancer cells using two independent shRNAs to knock down NUCB2 in SNU449 and Huh7 cells. Compared with the control group, NUCB2 protein levels were significantly reduced in the shRNA groups (Figure 2A). Colony-forming and EdU assays revealed that NUCB2 depletion led to a notable reduction in colony formation and cell proliferation (Figures 2B-E). The Transwell assay indicated that the migration degree of NUCB2-depleting cells was less than that of control cells (Figures 2F-G). Similar results were obtained in the sphere formation assay (Figures 2H-I).

**Figure 3:**
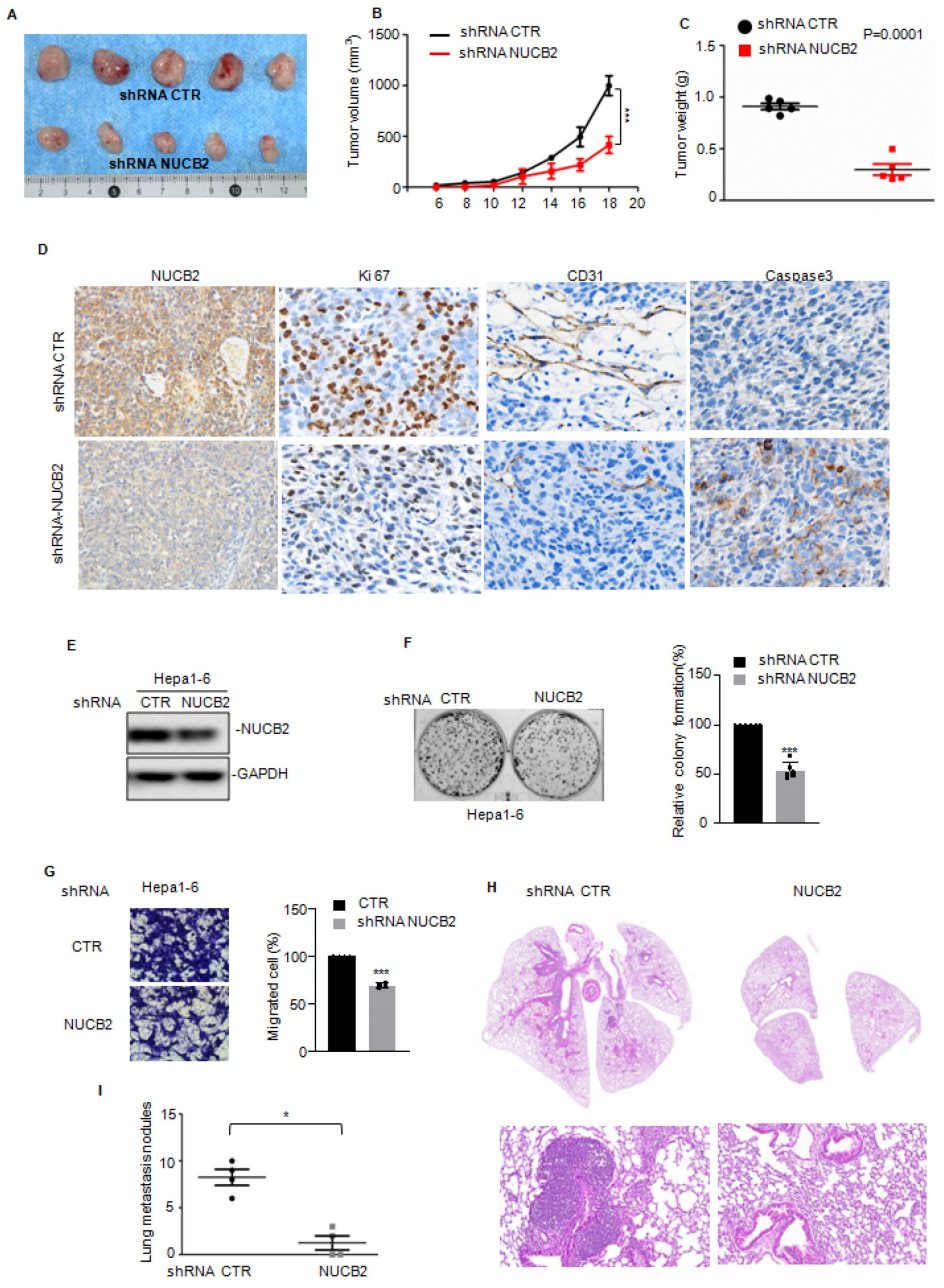
(A) Huh-7 cells with or without NUCB2 knockdown were injected into NOD/SCID female mice (n = 5). Representative xenograft tumor images. The calculation and analysis of tumor volume (B) and weight (C). (D) IHC analysis was conducted on tumor tissue sections using anti-NUCB2, anti-CD31, anti-Ki-67, and anti-cleaved caspase-3 antibodies. (E-G) NUCB2 was knocked down in Hepa1-6 cells. NUCB2 expression was detected by Western blotting. Cell growth and migration were assessed using colony formation and Transwell assays. (H-I) Administration of intravenous injection Hepa1-6 cells with or without NUCB2 depletion to C57BL/6J mice (n = 4). Images of HE staining and all groups of metastatic nodules were evaluated. The B, C, F, G, and I data were analyzed by Student's t-test, *p < 0.05, **p < 0.01, and ***p < 0.001.

To further verify the oncogenic role of NUCB2 *in vivo*, NUCB2-depleting Huh-7 cells were injected into NOD/SCID female mice, which discovered that NUCB2 depletion resulted in a significant reduction in tumor size and weight (Figures 3A-C). IHC analysis demonstrated that Huh-7 xenograft tumor tissues from NUCB2 depleting cells demonstrated decreased Ki-67 and CD31 positive cells and increased cleaved caspase 3 positive cells (Figure 3D).

Except that, we also assessed the effects of NUCB2 on mouse hepatoma cell proliferation and metastasis. Consistent with human HCC cells, NUCB2 knockdown inhibited cell growth and migration (Figures 3E-G). Subsequent xenograft models showed that inhibition of NUCB2 notably reduced the lung metastasis of Hepa1-6 cells (Figures 3H-I). Altogether, NUCB2 could facilitate HCC cell growth and metastasis *in vitro* and *in vivo*.

### NUCB2 facilitated PTGR1 expression in HCC cells

**Figure 4:**
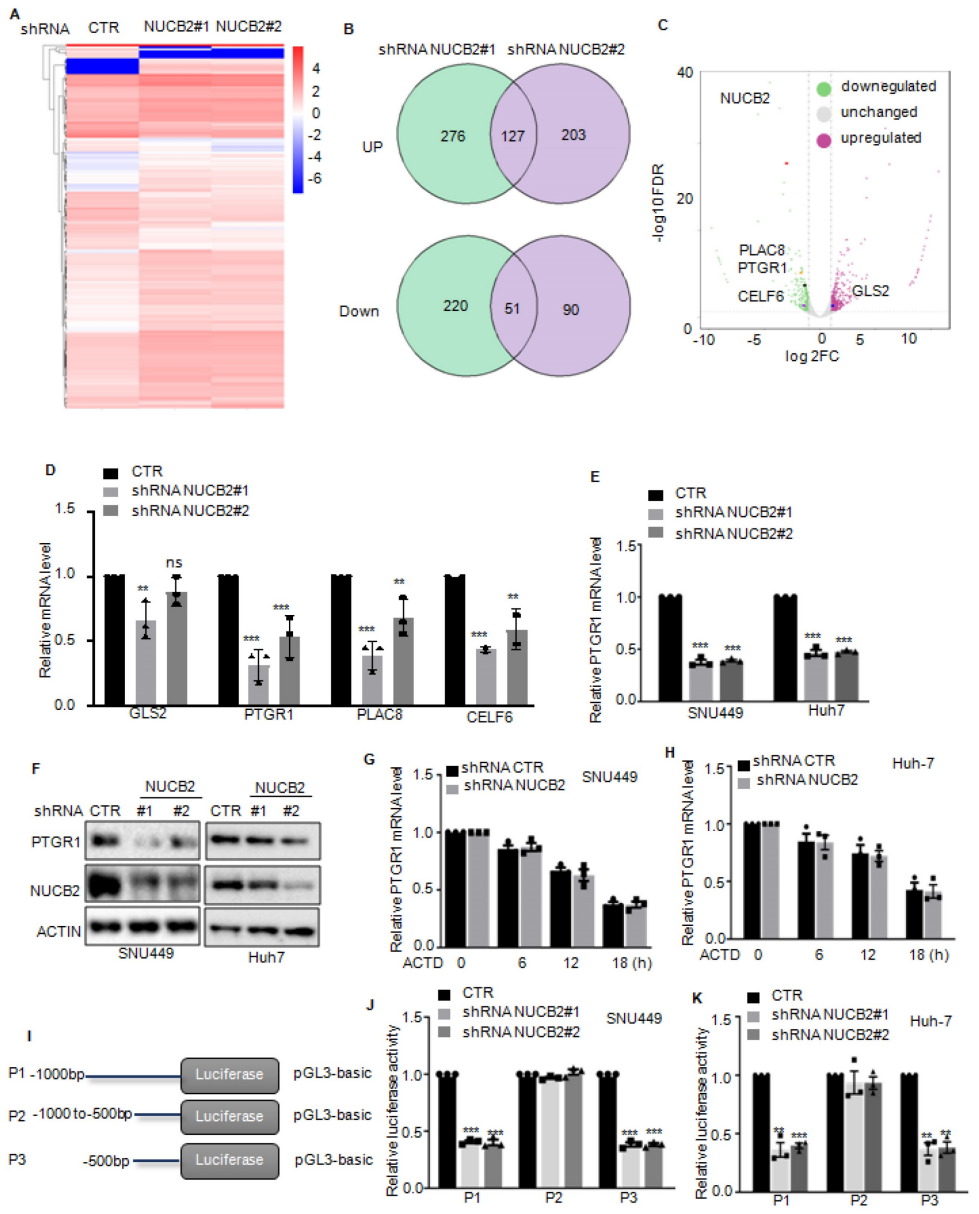
(A) Heatmap displaying the changed genes found through RNA sequencing analysis in SNU449 cells with or without NUCB2 knockdown. (B) Venny plots showing co-changed genes in two NUCB2 knockdown cells. (C) Alterations in various genes of NUCB2 knockdown cells shown in the volcano plot, with green indicating a decrease and red denoting an increase. (D) The expression levels of GLS2, PTGR1, PLAC8, and CELF6 in SNU449 cells with or without NUCB2 knockdown were detected by qRT-PCR. (E-F) PTGR1 mRNA and protein levels were detected by qRT-PCR and Western blotting in SNU449 and Huh-7 cells. (G-H) SNU449 and Huh-7 cells with or without NUCB2 depletion were treated with actinomycin D (ACTD) at the indicated times, and the mRNA levels of NUCB2 were detected by qRT-PCR. (I) Schematic illustration of pGL3-based P1/2/3 reporter constructs used in luciferase assays to examine PTGR1 transcriptional activity. (J-K) P1/2/3 and Renilla luciferase plasmid transfected into SNU449 and Huh-7 cells with or without NUCB2 depletion. The luciferase activity of PTGR1 was measured. The D, E, J and K data were analyzed by Student's t-test, *p < 0.05, **p < 0.01, and ***p < 0.001.

Furthermore, we uncovered the underlying molecular mechanism by which NUCB2 promotes HCC cell growth and metastasis via RNA sequencing analysis to ascertain the downstream genes of NUCB2 (Figure 4A). Overlapping the altered genes between shRNA NUCB2-1 and shRNA NUCB2-2, we obtained 127 upregulated and 51 downregulated genes (Figure 4B). Among the altered genes, GLS2, PTGR1, PLAC8, and CELF6 were apparently downregulated (Figure 4C). Next, we assessed GLS2, PTGR1, PLAC8, and CELF6 expression in NUCB2-depleting HCC cells and found that NUCB2 inhibition steadily and significantly decreased PTGR1 mRNA and protein levels (Figures 4D-F).

We analyzed the mRNA stability of PTGR1 in HCC cells with or without NUCB2 depletion to explore the molecular mechanism by which NUCB2 upregulated PTGR1 RNA and protein levels and found that inhibition of NUCB2 showed no effect on the mRNA stability of PTGR1in SNU449 and Huh-7 cells (Figures 4G-H). Subsequently, we introduced the promoter and different truncations of PTGR1 into the pGL3 vector, which we named P1/2/3. (Figure 4I). These plasmids were transfected into HCC cells, with or without NUCB2 inhibition. The promoter activity of PTGR1 was measured, which indicated that P1/3 activity was reduced by NUCB2 knockdown, whereas P2 exhibited a non-significant change (Figures 4J-K). These findings suggested that NUCB2 enhances PTGR1 expression in a transcription-dependent manner and that the region (-500 to -0 bp) is key for upregulating PTGR1 expression by NUCB2.

### NUCB2 promoted HCC cell proliferation and migration by upregulating PTGR1 expression

**Figure 5:**
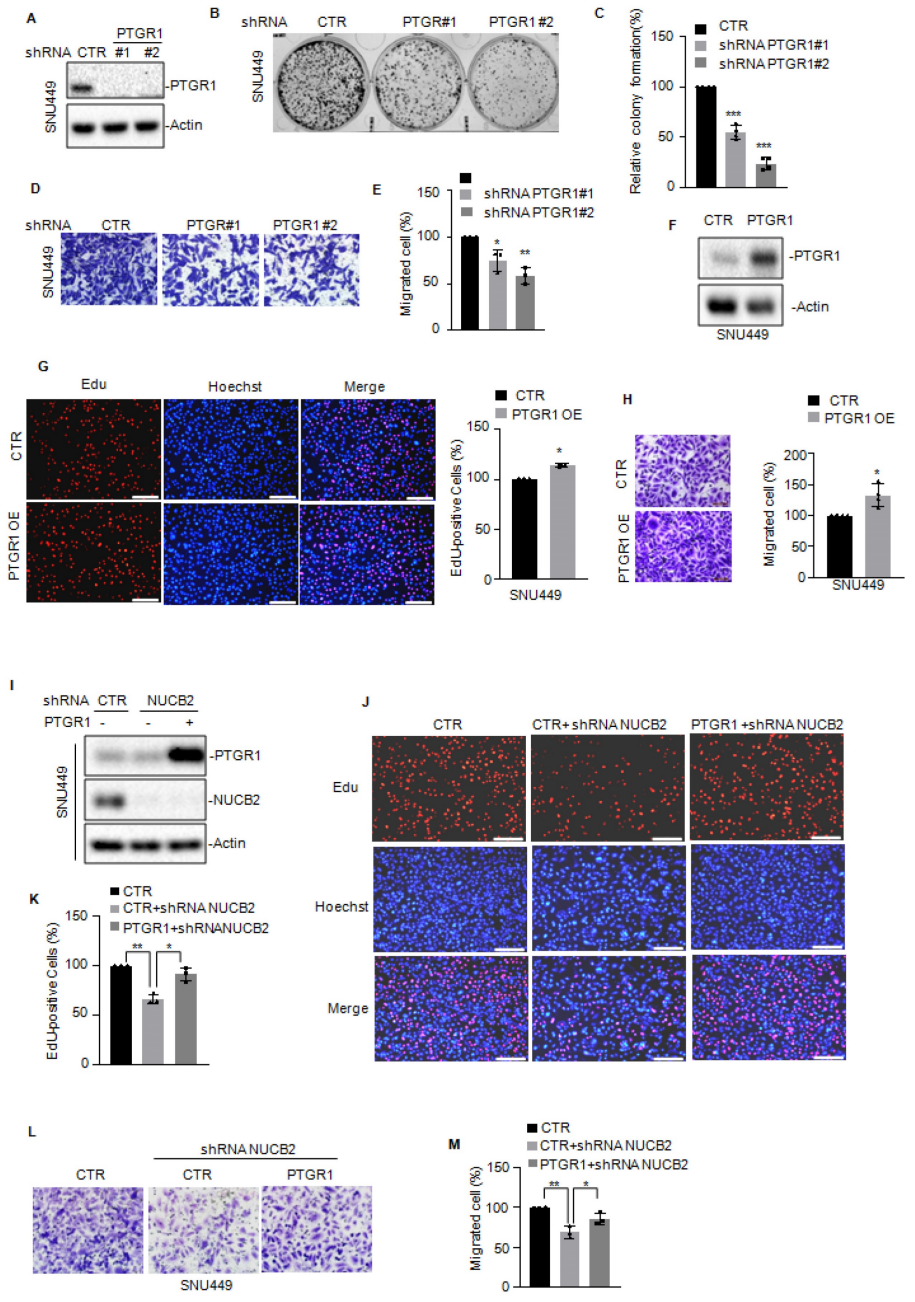
(A) PTGR1 was knocked down in SNU449 cells, and the expression of PTGR1 was detected by Western blotting. (B-C) The colony formation assay was used to investigate the effect of PTGR1 knockdown on SNU449 cell growth. (D-E) Transwell assay was used to measure the indicated cell migration. (F) PTGR1 was overexpressed in SNU449 cells, and the expression levels of PTGR1 were detected by Western blotting. (G) EdU assay was used to measure the effect of PTGR1 on cell growth. (H) Transwell assay was used to measure the impact of PTGR1 overexpression on cell migration. (I-K) PTGR1 was overexpressed in SNU449 cells with or without NUCB2 knockdown. Cell proliferation was detected by EdU assays. (L-M) Cell migration was analyzed by Transwell assay. The C, E, G, H, K, and M data were analyzed by Student's t-test, *p < 0.05, **p < 0.01, and ***p < 0.001.

To assess whether NUCB2 enhances cell proliferation and migration by elevating PTGR1, we first knocked down PTGR1 in SNU449 cells and assessed the effect of PTGR1 on cell proliferation and migration. As presented in Figures 5A-E, PTGR1 depletion suppressed cell proliferation and migration. In contrast, elevated PTGR1 levels enhanced cell proliferation and migration (Figures 5F-H). Then, we overexpressed PTGR1 in NUCB2-depleting cells and analyzed the protein levels of PTGR1 and NUCB2 using Western blotting (Figure 5I). The EdU and Transwell assay results indicated that increased PTGR1 reversed the suppressive effect of NUCB2 knockdown on cell proliferation and migration (Figures 5J-M). Taken together, these data implied that NUCB2 promotes the malignancy of HCC by increasing PTGR1 expression.

### NUCB2 suppressed E2F4 degradation

**Figure 6:**
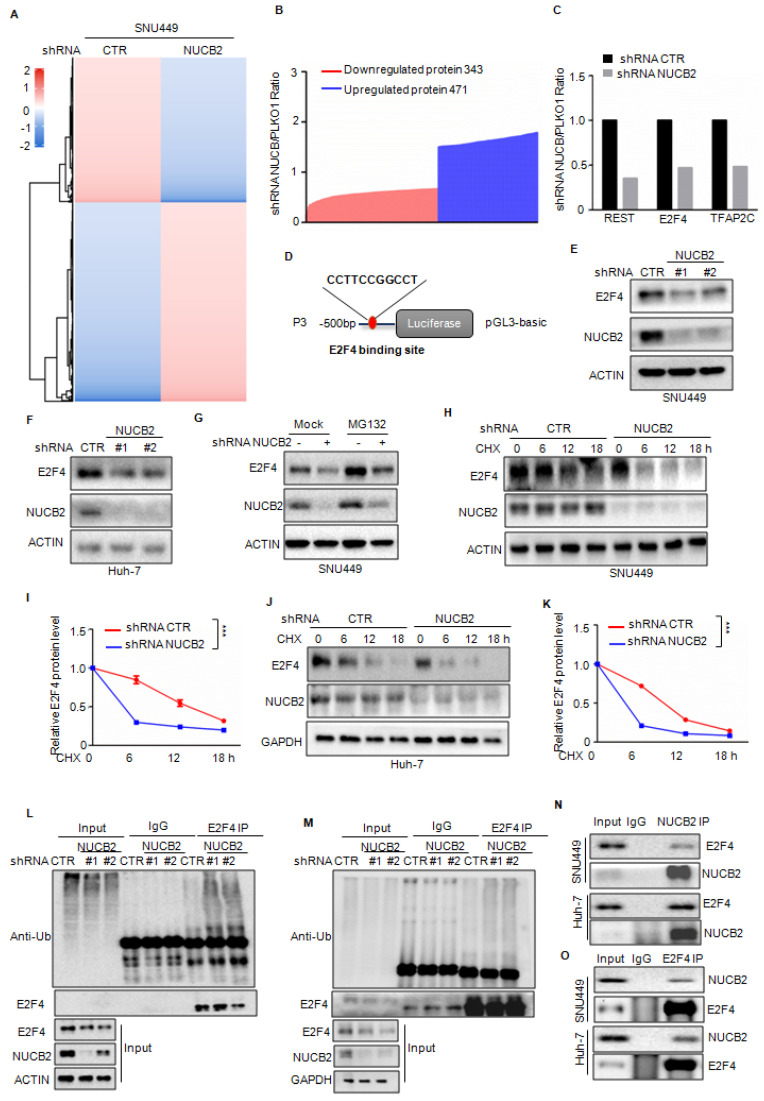
(A-B) label-free quantitative proteomics was used to identify the altered proteins in NUCB2 depleting or control cells. (C) The downregulated transcriptional factors are listed. (D) The positive binding site of E2F4 on the promoter of PTGR1 was obtained from the JASPAR database. (E-F) The protein levels of E2F4 in SNU449 and Huh-7 cells with or without NUCB2 depletion were analyzed by Western blotting. (G) SNU449 cells with or without NUCB2 depletion were treated with or without MG132 for 8 h, and the protein levels of E2F4 were detected by Western blotting. (H-K) SNU449 and Huh-7 cells with or without NUCB2 depletion were treated with cycloheximide (CHX) at the indicated times, and the protein levels of E2F4 were detected by Western blotting. (L-M) SNU449 and Huh-7 cells with or without NUCB2 knockdown were transfected with the indicated constructs and treated with MG132 for 8 h before collection. The whole-cell lysate was subjected to immunoprecipitation with an E2F4 antibody and Western blotting with an anti-Ub antibody to detect ubiquitylated E2F4. (N-O) SNU449 and Huh-7 cell lysates were subjected to immunoprecipitation with control IgG or anti-E2F4 or NUCB2 antibodies. The immunoprecipitates were detected via the indicated antibodies. The I and K data were analyzed by Student's t-test, *p < 0.05, **p < 0.01, and ***p < 0.001.

To better understand the downstream mechanism whereby NUCB2 transcriptionally upregulates PTGR1 in HCC, label-free quantitative proteomics was employed to search for altered proteins in NUCB2-depleting cells, and 343 downregulated and 471 upregulated proteins were obtained (Figures 6A-B). Among these altered proteins, we found that REST, E2F4, and TFAP2C transcription factors significantly decreased when NUCB2 was knocked down (Figure 6C). Based on the data in the Figures 4J-L, it was known that the promoter (-500 to 0 bp) of PTGR1 was essential for the upregulation of PTGR1 by NUCB2. Thus, the PTGR1 promoter (-500 to -0 bp) was inspected using the JASPAR database (https://jaspar.genereg.net), and the positive binding site of E2F4 was found on the region of the PTGR1 promoter (-500 to -0 bp) (Figure 6D). Accordingly, E2F4 was selected as the downstream regulating transcription factor, which may transcriptionally upregulate PTGR1 expression.

To valid the relationship between E2F4 and NUCB2, the expression levels of E2F4 were first detected in SNU449 and Huh-7 cells with or without NUCB2 knockdown, which showed that knockdown of NUCB2 exhibited no effect on E2F4 mRNA level; however, protein levels of E2F4 were reduced (Figures 6E-F and Supplementary Figures 1). Simultaneously, proteasome inhibitor MG132 treatment reversed the E2F4 decline by NUCB2 knockdown, suggesting that NUCB2 elevated E2F4 in a proteasome-dependent manner (Figure 6G). To further verify it, the stability of E2F4 was assessed, indicating that NUCB2 knockdown shortened the half-life of E2F4 in HCC cells (Figures 6H-K). The findings of this study also indicated that inhibition of NUCB2 elevated the ubiquitylation of E2F4 (Figures 6L-M).

Therefore, it was hypothesized that NUCB2 may interact with E2F4. To confirm the physical interaction between NUCB2 and E2F4, coimmunoprecipitation (Co-IP) assays were conducted in HCC cells. The results found E2F4 a true interacting partner of NUCB2 (Figures 6N-O). Our data suggested that E2F4 acts as a distinct substrate of NUCB2, and NUCB2 knockdown suppresses E2F4 expression in HCC cells.

### E2F4 is directly bound to the promoter of PTGR1 and transcriptionally upregulates PTGR1 in HCC cells

**Figure 7:**
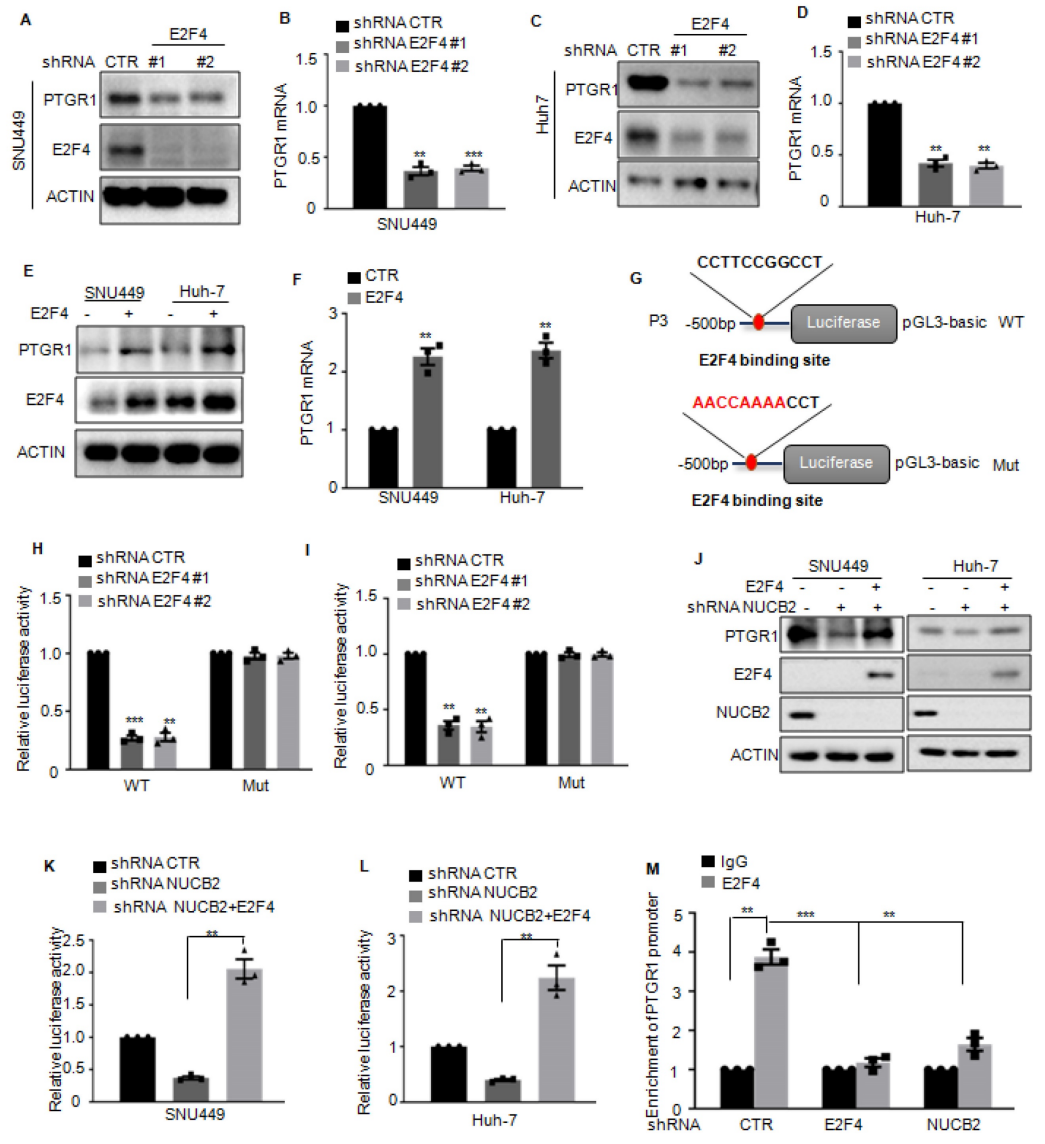
(A-D) The protein and mRNA levels of PTGR1 were analyzed by Western blotting and qRT-PCR in hepatocellular carcinoma cells with or without E2F4 depletion. (E-F) The protein and mRNA levels of PTGR1 were analyzed by Western blotting and qRT-PCR in hepatocellular carcinoma cells with or without E2F4 overexpression. (G) Schematic illustration of pGL3-based wild-type or mutant of E2F4 binding site on PTGR1 promoter constructs used in luciferase assays to examine PTGR1 transcriptional activity. (H-I) The wild-type or mutant of the E2F4 binding site on the PTGR1 promoter transfected into SNU449 and HepG2 cells with or without NUCB2 depletion. The luciferase activity was measured. (J) E2F4 was overexpressed in SNU449 and HepG2 cells with or without NUCB2 depletion. The protein levels of PTGR1 were detected by Western blotting. (K-L) The wild-type E2F4 binding site on the PTGR1 promoter transfected into the indicated cells. The luciferase activity was measured. (M) The E2F4 binding to the PTGR1 promoter in SNU449 cells with or without NUCB2 or E2F4 knockdown shown by ChIP analysis. Isotype-matched IgG was used as a negative control. The B, D, F, H, I, K, L, and M data were analyzed by Student's t-test, *p < 0.05, **p < 0.01, and ***p < 0.001.

To further elucidate the relationship between E2F4 and PTGR1, PTGR1 expression in HCC cells with or without E2F4 depletion was explored, which demonstrated that E2F4 suppression downregulated PTGR1 protein and RNA levels (Figures 7A-D). Conversely, E2F4 overexpression upregulated PTGR1 expression (Figures 7E-F).

To verify the transcriptional upregulation of PTGR1 by E2F4, we constructed the wild-type (WT) and the mutant (Mut) binding site of E2F4 into pGL3 plasmid (Figure 7G). These plasmids were then transfected into E2F4 depleting- SNU449 and Huh-7 cells, and the results of luciferase activity indicated that E2F4 suppression downregulated the luciferase activity of WT but not Mut (Figures 7H-I). To further determine whether NUCB2 upregulated PTGR1 expression relies on E2F4, we overexpressed E2F4 in SNU449 and Huh-7 cells with NUCB2 knockdown and found that E2F4 overexpression recovered the downregulation of PTGR1 protein levels by NUCB2 depletion (Figure 7J). Similarly, E2F4 overexpression abolished the reduction of PTGR1 promoter activity induced by NUCB2 knockdown (Figure 7K-L). In addition, the ChIP experiments showed that the promoter sequence of PTGR1 was enriched in anti-E2F4 immunoprecipitation. However, the enrichment of the PTGR1 promoter was impaired in E2F4 or NUCB2-depleted SNU449 cells (Figure 7M). In summary, the data indicated that E2F4 binds to the promoter of PTGR1, which induces its transcription, and NUCB2 enhances PTGR1 expression in an E2F4-dependent manner.

### Increased NUCB2 was positively associated with E2F4 and PTGR1 upregulation in human HCC

**Figure 8:**
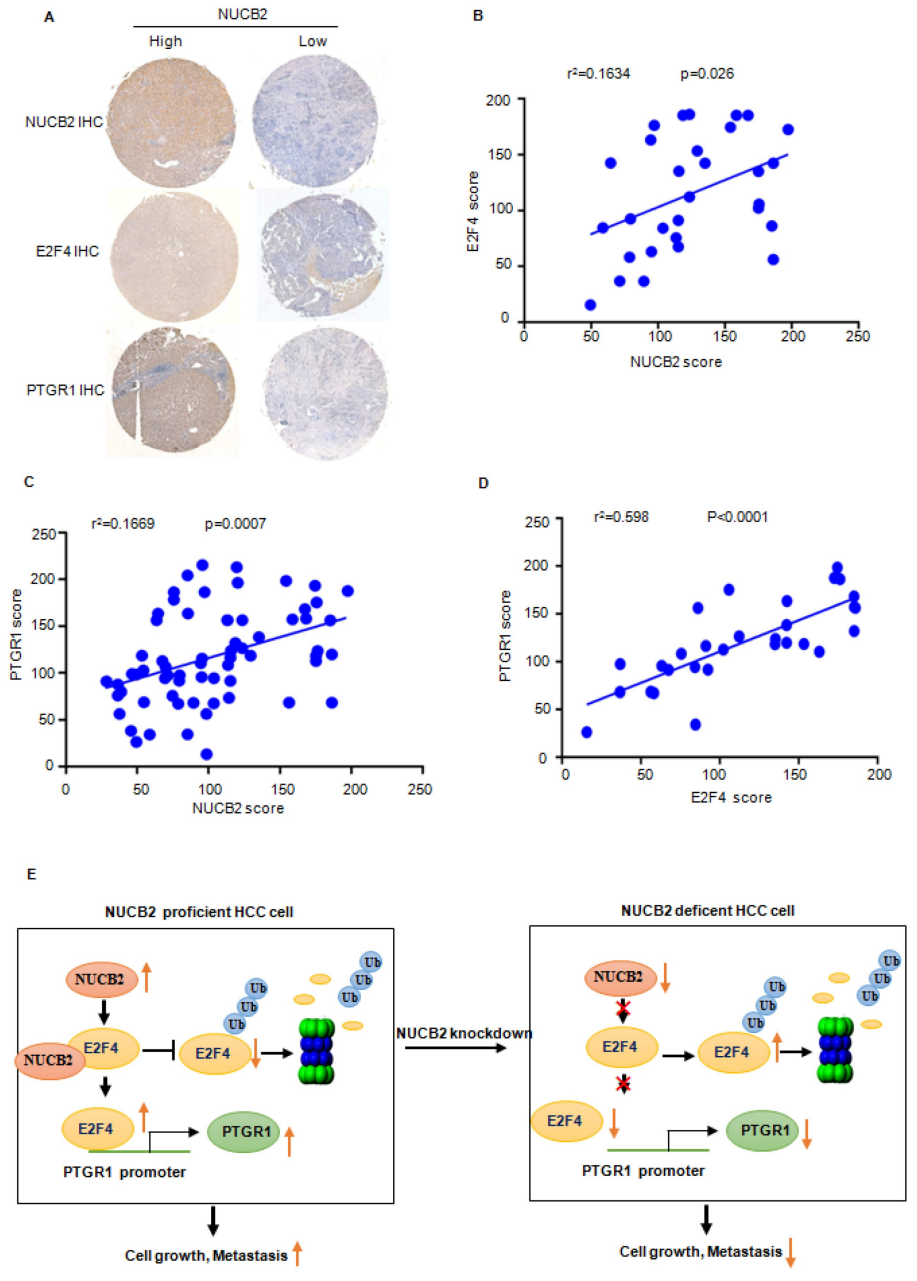
(A) Representative NUCB2, E2F4, and PTGR1 immunohistochemical staining microphotographs of the indicated hepatocellular carcinoma tissues. (B-D) The analysis investigated the association between the NUCB2, E2F4, and PTGR1 expression. Data were analyzed using Pearson's correlation. (E) Pattern diagram illustrating the relationship between NUCB2, E2F4, and PTGR1 in HCC.

To verify the truth of the NUCB2/E2F4/PTGR1 axis in HCC, the IHC staining and correlation analysis were performed to investigate the association between NUCB2, E2F4, and PTGR1. The results revealed that NUCB2 positively correlated with E2F4 and PTGR1 in HCC (Figures 8A-C). Likewise, the protein levels of E2F4 were also associated with PTGR1 expression in HCC (Figure 8D).

## Discussion

This study reported the oncogenic role of NUCB2 in HCC. NUCB2 acted as an interacting partner of E2F4, suppressing E2F4 degradation and enhancing its expression in HCC. Elevated E2F4 bound to the PTGR1 promoter enhanced its transcriptional upregulation, increasing cell proliferation and migration.

NUCB2, a member of the nucleobindin family, has been indicated to take part in many physiological processes, including food intake, energy metabolism, and regulation of the immune, cardiovascular, and endocrine systems^7^. Increasing evidence highlighted that NUCB2 is an important regulator in several cancers.

NUCB2 enhanced breast cancer metastasis via upregulating cholesterol synthesis^11^. In melanoma, NUCB2 overexpression suppressed ER stress-induced cell death^14^. The AMPK/TORC1/ZEB1 pathway is influenced by NUCB2, which is vital for cell metastasis^12^. Recently, it was found that NUCB2 enhanced osteosarcoma immune escape by elevating CXCL8 expression^22^. Correspondingly, this study demonstrated an oncogenic function of NUCB2 in HCC, that NUCB2 was notably increased in HCC, and that high expression of NUCB2 was closely correlated with poor prognosis. NUCB2 knockdown decreased cell proliferation and migration *in vitro* and *in vivo*.

The subsequent RNA sequencing analysis was performed to uncover the mechanisms by which NUCB2 promoted HCC progression. We identified that NUCB2 depletion markedly decreased PTGR1 expression. Multiple studies reported the involvement of PTGR1 in tumor development^23^. Elevated PTGR1 increased the prostate cancer cell resistance to metformin and promoted cell proliferation^24^, ^25^. In breast cancer, PTGR1 reduced breast cancer stem cells and reduced cell proliferation^26^, ^27^. However, PTGR1 is reported to be downregulated, and a decrease in PTGR1 correlated with the poor prognosis in uterine corpus endometrial carcinoma, ovarian cancer, and kidney renal clear cell carcinoma^23^. In HCC, PTGR1 was upregulated and facilitated cell growth and resistance to oxidative stress in rat hepatocarcinogenesis^28^-^30^. Importantly, our study showed that NUCB2 elevated the activity of the PTGR1 promoter and promoted its transcription. Elevated PTGR1increased HCC cell proliferation and migration.

NUCB2 is not a transcription factor. To explore the mechanism of NUCB2-enhanced PTGR1 transcription, label-free quantitative proteomics was employed to identify the altered transcription factors when NUCB2 was knocked down. We found that E2F4 was a potential transcription factor that was downregulated in NUCB2-depleting cells. E2F4 is an E2F family transcription factor that is crucial for cell cycle regulation^31^. In HCC, E2F4 was significantly increased and promoted cancer progression via transcriptionally upregulating CDCA3 expression^32^.

E2F4 is an unstable protein, and proteasome degradation is a main modality in regulating E2F4 expression and function in cancers^33^, ^34^. E3 ligase TRIM33 interacted with E2F4, facilitating recovery of DNA synthesis^35^. USP2 could remove the ubiquitination of E2F4 and enhance its expression. This study found that NUCB2 interacted with E2F4 and suppressed its degradation^36^. Elevated E2F4 bound to the PTGR1 promoter and induced PTGR1 expression in HCC. Consequently, NUCB2 facilitated PTGR1 transcription via elevating E2F4 expression; however, the mechanism of NUCB2 upregulation in HCC and the precise mechanism of regulatory action between NUCB2 and E2F4 remains unclear and should be elucidated in further work.

## Conclusion

Overall, the findings of this study suggested that NUCB2 is expressed aberrantly in HCC. Elevated NUCB2 promotes tumor progress *in vitro* and *in vivo*. In addition, NUCB2 interacts with E2F4 and inhibits its degradation, resulting in the transcriptional upregulation of PTGR1 (Figure 8E). In conclusion, NUCB2 is a possible therapeutic target to elevate the survival rate of HCC patients.

## Supplementary Material

Supplementary figure.

## Abbreviations

HCC: hepatocellular carcinoma
NUCB2: nucleobindin 2
PTGR1: prostaglandin reductase 1
E2F4: E2F transcription factor 4
Co-IP: coimmunoprecipitation
ChIP: Chromatin immunoprecipitation
