# Supplementary material for: NUCB2 promotes hepatocellular carcinoma cell growth and metastasis by activating the E2F4/PTGR1 axis
Source: Int J Biol Sci. 2024 Sep 3;20(12):4767–80. doi: 10.7150/ijbs.97861 (PMC11414389; doi:10.7150/ijbs.97861)

Supplementary Figure 1 The RNA levels of E2F4 was analyzed by qRT-PCR in SNU449 and Huh-7 cells with or without NUCB2 depletion.

Supplementary Figure 1

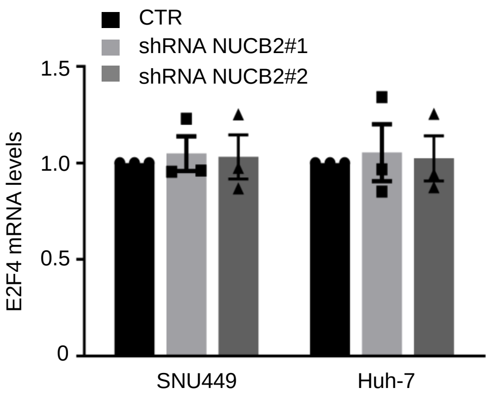

Supplement: Supplementary file 1 — Supplementary figure. [file ijbsv20p4767s1.pdf]
